# Training Needs of Manitoba Pharmacists to Increase Application of Assessment and Prescribing for Minor Ailments into Practice: A Qualitative and Quantitative Survey

**DOI:** 10.3390/pharmacy6030082

**Published:** 2018-08-04

**Authors:** Brenna Shearer, Sheila Ng, Drena Dunford, I fan Kuo

**Affiliations:** 1 College of Pharmacy, Faculty of Health Sciences, University of Manitoba, Winnipeg, MB R3E 0T5, Canada; drena.dunford@umanitoba.ca (D.D.); i.kuo@umanitoba.ca (I.f.K.); 2 Independent Researcher Pharmacists Manitoba, Winnipeg, MB R3C 4H1, Canada

**Keywords:** pharmacists, prescribing, professional development, minor ailments, continuing education needs, survey

## Abstract

Current literature demonstrates the positive impact of pharmacists prescribing medication on patient outcomes and pharmacist perceptions of the practice. The aim of this study was to understand the factors affecting prescribing practices among Manitoba pharmacists and identify whether additional training methods would be beneficial for a practice behavior change. A web-based survey was developed and participation was solicited from pharmacists in Manitoba. Descriptive statistics were calculated to summarize the frequency of demographic characteristics. Chi-square tests were used to explore possible correlations between variables of interest and thematic analysis of qualitative data was completed. A total of 162 participants completed the survey. The response rate was 12.3%. Of those who had met the requirements to prescribe, none were doing so on a daily basis and 23.5% had not assessed or prescribed since being certified. Respondents identified the top barriers for providing this service as a lack of sufficient revenue and a lack of time. Qualitative analysis of responses identified additional barriers including a limiting scope and inadequate tools. Approximately half (54.4%) of respondents expressed that additional training would be of value. The themes identified from the survey data suggest that practice-based education would help pharmacists apply skills. In addition, expansion of prescribing authority and strategies addressing remuneration issues may help overcome barriers to pharmacists prescribing within Manitoba.

## 1. Introduction

Pharmacy practices have expanded in some regions of Canada to include the authority to prescribe medications for a variety of conditions [1]. Legislation and regulation governing the scope of pharmacy practices are a provincial/territorial jurisdiction in Canada and, therefore, varies substantially across the country. Pharmacists in the province of Manitoba were granted regulatory authority to assess and prescribe medication for ambulatory ailments due to the passing of the Pharmaceutical Act and Regulations in January 2014. The Pharmaceutical Regulations enable pharmacists to prescribe any Schedule II or III drug listed in the National Association of Pharmacy Regulatory Authorities (NAPRA) manual (non-prescription) and Health Canada approved medical devices. In addition, authorization to prescribe specific medications for select self-limiting conditions and smoking cessation (list specified in the Pharmaceutical Regulations Schedule III) can be obtained by any licensed pharmacist with successful completion of an education program approved by the provincial regulatory body known as the College of Pharmacists of Manitoba. The authorization to prescribe a drug for self-limiting conditions requires the completion of a web-based independent study program with an exam administered by the College of Pharmacists of Manitoba [2].

In the 20 months following the expansion of this scope of practice, 425 pharmacists had successfully completed the requirements of the Manitoba Certification for Authorization to Prescribe a Drug Included in the Schedule 3 to the Pharmaceutical Regulation for Self-Limiting Conditions (College of Pharmacists of Manitoba, personal email, 3 September 2015). Two hundred and twenty-five (225) pharmacists have successfully completed the Manitoba Certification to Prescribe a Drug Included in Schedule 3 to the Pharmaceutical Regulations for Smoking Cessation (College of Pharmacists of Manitoba, personal email, 3 September 2015). With more than 1501 licensed pharmacists in Manitoba, the uptake by pharmacists has been slow.

Across Canada there is variation in publicly funded and third party health insurance benefits to compensate for pharmacist initiated assessment and prescribing services [3]. While pharmacists in Manitoba have one of the most comprehensive scopes of practice, they have one of the most limited compensation models along with one of the most detailed training requirements [1,4]. Compensation frameworks may play a pivotal role in pharmacist decision-making regarding the application of these services into practice especially in Manitoba where a publicly funded framework for services has not kept pace with the rest of Canada [4]. However, a growing body of literature demonstrates the positive impact on patient outcomes of pharmacist prescribing medication, pharmacist perceptions of the practice, and practice changes [3].

Knowledge translation literature describes successful integration of knowledge into practice as a key factor to improve practitioner confidence [5]. Research on practice changes has demonstrated that significant factors for success include pharmacist’s perceptions about factors influencing uptake of prescribing practices, identifying positive practice settings, confidence, and training programs incorporating both the knowledge and the ability to apply knowledge [3,6,7,8,9]. Some studies have indicated that pharmacists preferred live training programs and evaluated these as the most valuable for improving confidence [10,11]. In addition, communication and counseling skills are stated as important components for reducing practice barriers and ensuring confidence above and beyond continuing education programs [6,12]. Pharmacist prescribing education programs vary widely across the country, which may affect the consistency of application within the profession [1].

There has been no research evaluating how many Manitoba pharmacists with prescribing certifications are actively practicing this expanded scope of practice, what gaps exist between continuing professional development and application to the practice, what the training and education needs of pharmacists are to successfully apply this knowledge into practice, and what factors influence pharmacists to engage in prescribing authority education. The information gathered can inform and impact the development of effective continuing education programs that address pharmacists’ needs. The aim of this research study is to understand the factors impacting prescribing authority practice among Manitoba pharmacists and identify whether additional training methods would be beneficial for practice behavior change.

## 2. Methods

### 2.1. Survey Design

An anonymous web-based survey was developed, which includes a variety of question modalities such as multi-select questions, single-select questions, dichotomous answers, rank ordering, and open-ended free text. Questions included demographic information, the status of Certification in Prescribing for Self-Limiting Conditions, factors affecting adoption of prescribing knowledge into the practice, and attitudes regarding training needs to increase the provision of prescribing drugs into clinical practice. A literature search was completed to identify previous surveys, which evaluated pharmacist’s continuing education needs and preferences. Relevant resources were identified [6,7,11,12,13,14,15,16,17]. The research team reviewed the literature for relevant questions, themes, and modified questions to pertain specifically to pharmacist’s continuing education needs and preferences for prescribing drugs for self-limiting conditions in Manitoba. Additional questions were developed to assess the demographic and status of certification specific to Manitoba.

The survey contained three question streams that filtered participants based on their status of completion of the self-study program required for prescribing drugs for self-limiting conditions. The status was broken down into four categories, which includes: (1) pharmacists that have completed the self-study program, (2) pharmacists in progress of completing the self-study program, (3) pharmacists who had not started but plan to initiate the self-study program, and (4) pharmacists with no plans to start the self-study program. All streams answered four demographic questions and one status of certification question. Participants in all categories answered a question regarding either experienced or anticipated barriers as well as four questions regarding continuing education preferences and needs. Wording was modified within the questions to reflect participants’ current prescribing status. Participants who had completed the self-study program (category 1) answered three additional questions regarding the frequency of and fees charged for prescribing.

The draft survey was first piloted among eight pharmacist volunteers from the Pharmacists Manitoba Board of Directors. The pilot survey resulted in no changes. The final survey was administered through FluidSurveys™.

### 2.2. Recruitment and Data Collection

Recruitment of participants took place over an eight-month period from June 2016 to January 2017. The non-probability convenience sampling method was utilized. Email databases from all provincial pharmacy organizations including the Canadian Society of Hospital Pharmacists (CSHP) Manitoba Branch, the College of Pharmacists of Manitoba (CPhM), and Pharmacists Manitoba were used to distribute the survey link and information to capture all pharmacists licensed in Manitoba. The project received approval by the University of Manitoba Health Research and Ethics Board (approval number HS19741) and all participants provided informed consent prior to data collection.

### 2.3. Statistical Analysis

Descriptive statistics were calculated to summarize the frequency of demographic characteristics for overall survey respondents and subgroups of survey respondents. Chi-square tests were used to explore possible correlation between variables of interest (i.e., barriers, service fees, and preferences for training) and demographics for each group. Those who were in the process of completing or planning on initiating the self-study program answered the same set of survey questions and were treated as the same group in the analyses. All statistical analyses were carried out in the statistical software SAS^®^ 9.4 (SAS Institute; Carey, NC, USA). Qualitative analysis of free text responses was also completed using FluidSurveys™. In addition, the research team completed realist thematic analysis to identify the main themes to summarize collective responses using word repetitions and key-words-in-context methods [18].

## 3. Results

### 3.1. Demographics of Survey Respondents

During the eight-month recruitment period, 185 participants consented to the research study. Of those, 162 completed the online survey (completion rate 87.6%). The survey response rate was estimated to be 12.3% based on the total number of pharmacists invited to participate in the study (185 respondents out of 1501 licensed pharmacists). The majority of the respondents practiced in the retail setting (90.7%), worked in the Winnipeg Regional Health Authority (WRHA) catchment (52.5%), and were predominantly employed in a full-time position (75.3%). Most respondents had been in practice for more than 15 years (45.7%), which was followed by those in practice for six to 15 years (32.1%), and those in practice for fewer than six years (22.2%). The characteristics of survey respondents are described in Table 1.

Of the 162 respondents, 115 (71.0%) reported having completed the self-study program for assessments and prescribing for minor ailments. Nine individuals (5.6%) were in progress of completing the self-study program, 23 (14.2%) respondents had yet to start but were planning to initiate the program, and 15 (9.3%) pharmacists expressed having no plans to start the program. There were no significant differences in demographics between the groups with the exception of pharmacists in the hospital setting being less likely to initiate a self-study program for assessments and prescribing for minor ailments than pharmacists in the retail setting (62.5% vs. 6.8%, *p* < 0.0001).

### 3.2. Assessment and Prescribing Skills for Self-Limiting Conditions in the Practice Setting

Amongst the 115 respondents who have completed the self-study program, none were providing assessment and/or prescribing services for minor ailments on a daily basis and 27 (23.5%) had not assessed or prescribed for minor ailments since receiving their certification. Of the 88 pharmacists who had provided minor ailments assessment or prescribing services, 52 (59.1%) reported having done so 1 to 5 times in the past 30 days, and 48 (54.5%) described having charged a fee for their services with a median amount of $20. The employment status was found to be significantly correlated with charging service fees for minor ailments assessment and prescribing where those not in a full-time employment position were more likely to have charged a fee (84.6% vs. 51.4%, *p* = 0.03). No correlation was found between charging a clinical service fee and other demographic characteristics.

### 3.3. Perceived Barriers for Applying Assessment and Prescribing Skills

When asked about barriers, the majority (83.5%) of the respondents certified to assess and prescribe drugs for minor ailments reported having encountered issues in providing such a service (Table 2). The top barriers identified were ‘lack of sufficient revenue attached to expanded role’ (26.2%), ‘lack of time at work’ (23.5%), and lack of patients presenting with minor ailments (11.9%). The region of employment was found to be correlated with reporting barriers where those in the WRHA catchment were more likely to have responded to having barriers than those outside of the WRHA area (90.9% vs. 76.7%, *p* = 0.04). No correlation was found between barriers and other respondent characteristics.

Of the 32 respondents who were in the process of completing the self-study program or were planning to initiate the program, 20 (62.5%) reported anticipating barriers that would prevent them from applying the assessment and prescribing skills for minor ailments in their practice. The top barriers identified were similar to the group who had been certified with ‘lack of sufficient revenue attached to expanded role’ (29.8%) and ‘lack of time at work’ (31.9%) as the most common barriers. The potential obstacles identified also included a ‘lack of confidence in skills’ (8.5%) and a ‘lack of support from management/head office’ (8.5%). No correlation was found between anticipating barriers and any of the demographic characteristics.

A minority of 15 respondents expressed having no plans to start the self-study program for the assessment and drug prescribing for self-limiting conditions. When asked about specific barriers that prevented them from taking on the expanded practice role, many perceived a ‘lack of sufficient revenue attached’, a ‘lack of time at work,’ and a ‘lack of motivation to take on new responsibilities’ as factors deterring them from undertaking the program.

**Figure 1:**
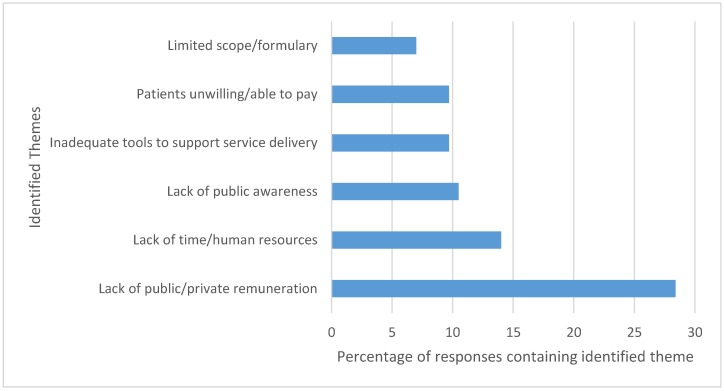
Themes identified from thematic analysis of open-response comments of pharmacists’ experience and perspectives of providing assessment and drug prescribing services.

The survey also encouraged respondents to provide additional comments on their experience and their perspectives of providing assessment and drug prescribing services for self-limiting conditions. Some common themes that emerged from these open-ended questions included a lack of public and private payer remuneration for services, a limiting scope of practice and prescribing formulary, insufficient public awareness of services, and an absence of adequate documentation and decision-making tools (see Figure 1).

### 3.4. Preferences for Continuing Education

Respondents who have completed the self-study program, were in the process of completion, or were planning to initiate the program were also asked whether they felt additional training and continuing education would improve their application of assessment and drug prescribing services for minor ailments. Approximately half (54.4%) of the 147 respondents expressed that additional training would be of value in applying their expanded role with 29.3% and 16.3% respectively responding no or unsure for the benefit of additional training in this area. Attitudes towards additional training did not correlate with demographic characteristics. Respondents were split between the types of education opportunity with 46.2% and 53.8% preferring self-guided study and live sessions, respectively. Internet-based written materials were the preferred type of self-guided training/continuing education format while live lectures, hands-on workshops, and topic-specific short seminars were the top ranked medium for live training. There was no correlation with years of practice and type of education preferred.

## 4. Discussion

This is the first survey to investigate the factors affecting the prescribing authority practice among Manitoba pharmacists and identify whether an additional training method would be beneficial for practice behavior changes. While the response rate to the survey was less than desired, the information gathered provides valuable insight into pharmacist preferences and perceptions about educational needs and barriers affecting the assessment and prescribing scope of practice.

The web-based survey response rate was 12.3%. Johnson and Wislar and Dillman have identified declining response rates to surveys in the past few decades [19,20]. For this study, the sample size needed to achieve a 95% confidence level with a confidence interval of 4 would have required 433 participants (28% response rate). Research has identified lower response rates from web-based survey methodology even though there is a growing prevalence as a preferred methodology due to the ease of data collection and analysis, faster response time, and lower cost [8,21,22]. Web-based surveys with low response rates are consistent in the literature, have been identified as reliable for evaluation, and are not restrictive to the generalizability of findings [23,24,25]. Furthermore, web-based survey response rates of less than 10% have been reported as reliable for evaluation [22,26,27,28]. For survey response rates, Johnson and Wislar state that “there is no scientifically proven minimally acceptable response rate” [19] (p. 1805).

Barriers identified in this study for providing assessment and prescribing services for minor ailments included lack of sufficient revenue, lack of time, lack of support from employers, and lack of eligible patients requiring the services. This is consistent with findings of research related to medication assessment services in other Canadian settings. Isenor et al. described time, staffing, and reimbursement as barriers identified to fully adopting prescribing. Guirguis et al. described limitations in the practice environment including staffing [29,30]. While pharmacists are one of the most accessible primary care health care providers, based on the number of patient visits compared with primary care physicians, a limited range of prescribing authority combined with lack of appropriate remuneration potentially impacts patient perception of the pharmacist’s role and the pharmacist’s full adoption of services into practice [31]. Previous research supports these research findings that identify pharmacist’s perceived barriers to greater adoption of services into practice to include a lack of support from employers, a lack of appropriate remuneration, and a need for personalized education and practice as factors affecting the application of new scopes of practice [9,10,32].

Although to a lesser extent, lack of confidence in skills was another barrier identified by some pharmacists. Previous research of allied health professionals with prescribing responsibility identified “personal anxiety undermining confidence to prescribe” as a theme affecting the continuing professional development needs of these prescribers [33]. Clinical uncertainty was not a major barrier identified in our study. Therefore, continued professional development may need to focus on strategies to increase confidence that extend beyond clinical content. Further research to explore the rationale for lack of confidence may be beneficial.

This study highlighted factors for understanding the continuing education needs of pharmacists to enhance adaptation of expanded prescribing skills into practice as an important component for understanding behavior and practice changes. Research has highlighted a strong relationship between the community pharmacy organizational culture (such as cultural values for innovation, competitiveness, and social responsibility) with a pharmacist provision of expanded scope of practice services [34].

While prior research added to the knowledge of factors affecting education participation and environmental factors impacting the adoption into practice, this study builds on that knowledge by focusing on pharmacist education preferences as an additional key element for improving the application of these skills into practice. Previous research has ascertained that pharmacists undertake continuing education based on factors including achieving licensure requirement, relevance to personal interest, and self-improvement [35].

Continuing education preferences of Manitoba pharmacists was consistent with findings from previous research studies, which indicated that training preferences of pharmacists was highest for live support programs [10,11]. While 54.4% of our respondents expressed value in additional training to support application of expanded roles, respondents were split between self-guided study and live sessions [9,11,12,13,14]. In Manitoba, a 15-hour online training module reflective of the prescribing authority range is available from the provincial regulatory body. In addition, an 8-hour continuing education in-person workshop has been jointly offered by the university and provincial advocacy body to enhance the theory-based learning with low uptake in the courses offered. Continuing education (CE) courses of self-study, distance learning, and live CE programs are most common yet less effective in improving clinical practice behavior while interactive workshops are the most favorable format to enhance the translation of CE learning into practice [13,14]. To improve the adoption of new skills into practice, Ontario pharmacists identified limitations with existing continuing education resources and identified preferences for individual practice support [9].

The findings of this study indicate that, in Manitoba, a blend of in-person education and support directly within the pharmacy along with employer education and integration may enhance opportunities for growth and adoption of new scopes of service by pharmacists and the pharmacy business. Alternative options for reimbursement beyond public funding may be required as a component of business model development for services. Since this study identified a limited prescribing scope as a barrier, increasing the pharmacist scope of prescribing authority in Manitoba may also provide additional interest by pharmacists and employers, increase visibility of the role of the pharmacist among patients and other health care providers, and open the door for constructive dialogue with government and private funders about how pharmacists can advance health care system accessibility.

### Strengths & Limitation

The strengths of this research include a specific focus on perceptions of pharmacists regarding education preferences and barriers to implementing patient assessment and drug prescribing skills for minor ailments in one province of Canada. In addition, this research evaluates the differences in perceptions between pharmacists who have completed, are in the process of completing or intending to complete certification, and those with no intention to gain additional certification. Strong interest and support from the provincial regulatory body and professional associations is evident from the distribution of the survey to all licensed pharmacists in the province through their regular electronic communications. Pharmacists Manitoba in partnership with the College of Pharmacy, University of Manitoba share a common goal of advancing the pharmacist’s professional practice. The survey was supported by all provincial organizations involved with licensing pharmacists and advocating for pharmacists, which increased the reach to pharmacists regardless of where they practice or the type of practice location. The information gathered in this research survey provides valuable knowledge to support the development of continuing education programs to enhance the application of practice skills in pharmacies.

A limitation of this study is the use of a web-based survey alone to capture pharmacist perceptions and a lower than desired response rate. Personalized invitations to complete the survey to stratified groups of pharmacists, those who have completed certification, and those who have not may have bolstered response rates by each group and provided more robust findings for evaluation and analysis. This study is limited to Manitoba pharmacists and, therefore, may have limited application to a national audience. However, the results confirm that perceptions of educational needs and barriers to practice application are consistent with the research literature. In spite of the specific education requirements for prescribing authority in Manitoba [12], pharmacist perceptions about barriers to the implementation of skills into practice are also consistent with the research literature.

This study lacked an evaluation of employer-specific perceptions of current and expected education requirements to support the patient assessment and drug prescribing services. A comparison of employee and employer expectations of skills post certification may provide insight as to what employers would support to advance the integration of new services.

## 5. Conclusions

In order to attain the optimal outcomes from pharmacists prescribing medication for minor ailments in Manitoba, continuing education that addresses the needs of pharmacists, reduces perceived barriers, and is delivered in an effective manner is required. As highlighted by the findings of this research study, pharmacists in Manitoba share similar concerns, issues, and education support needs as those in other parts of the country. The themes identified for continuing education suggest that personalized and practice-based education would help pharmacists apply these skills into practice. Educational programming, which addressed barriers identified by Manitoba pharmacists in providing prescribing services, may be beneficial. In order to address identified barriers, it could be beneficial to incorporate prescribing into a busy practice site (overcoming time management and human resource barriers) as well as include strategies to promote pharmacy services and development and implementation of fee-for-service models. In addition to education support for pharmacists, employers should direct more support for the implementation of services as well as strategies to overcome remuneration issues and mechanisms to expand prescribing authority in order to motivate the public and funders to utilize pharmacist’s knowledge and skills to their full scope.

## Figures and Tables

**Table 1:** Characteristics of survey respondents (*n*, %).

	Overall (*n* = 162)	Self-Study Completed (*n* = 115)	Self-Study in Progress (*n* = 9)	Self-study not Started but Plan to Initiate (*n* = 23)	No Plans to Start Self-Study (*n* = 15)
**Number of years practicing as pharmacist**					
<6 years	36 (22.2%)	29 (25.2%)	1 (11.1%)	5 (21.7%)	1 (6.7%)
6–15 years	52 (32.1%)	33 (28.7%)	2 (22.2%)	7 (30.4%)	10 (66.7%)
>15 years	74 (45.7%)	53 (46.1%)	6 (66.7%)	11 (47.8%)	4 (26.7%)
**Employment status**					
Full time	122 (75.3%)	94 (81.7%)	5 (55.6%)	14 (60.9%)	9 (60.0%)
Part time	27 (16.7%)	16 (13.9%)	3 (33.3%)	4 (17.4%)	4 (26.7%)
Casual or on leave	12 (7.4%)	4 (3.4%)	1 (11.1%)	5 (21.7%)	2 (13.3%)
**Primary area of employment**					
Retail/community	147 (90.7%)	111 (96.5%)	6 (66.7%)	20 (87.0%)	10 (66.7%)
Hospital	8 (4.9%)	1 (0.9%)	1 (11.1%)	1 (4.3%)	5 (33.3%)
Government or academia	3 (1.9%)	2 (1.8%)	1 (11.1%)	0	0
Other	3 (1.9%)	1 (0.9%)	1 (11.1%)	1 (4.3%)	0
**Location of employment by health region**					
Winnipeg (Churchill)	85 (52.5%)	55 (47.8%)	6 (66.7%)	12 (52.2%)	1 (6.7%)
Interlake Eastern	16 (9.9%)	12 (10.4%)	1 (11.1%)	3 (13.0%)	0
Northern	5 (3.1%)	3 (2.6%)	0	1 (4.3%)	1 (6.7%)
Prairie Mountain Health	25 (15.4%)	19 (16.5%)	1 (11.1%)	4 (17.4%)	1 (6.7%)
Southern Health-Sante Sud	22 (13.6%)	20 (17.4%)	0	2 (8.7%)	12 (80.0%)

**Table 2:** Barriers identified by respondent subgroups (*n*, %).

	Self-Study Completed (*n* = 115)	Self-Study in Progress/Plan to Initiate (*n* = 32)	No Plans to Start Self-Study (*n* = 15)
**Encountered barriers in applying assessment and prescribing skills for minor ailments**			
Yes	96 (83.5%)	--	--
No	19 (16.5%)	--	--
**Anticipated encountering barriers in applying assessment and prescribing skills for minor ailments**			
Yes	--	20 (62.5%)	--
No	--	12 (37.5%)	--
**Identified barriers to taking on expanded role of applying assessment and prescribing skills for minor ailments**			
Yes	--	--	14 (93.3%)
No	--	--	1 (0.06%)
**Specific barriers identified (*n*, %)**			
Lack of sufficient revenue attached to expanded role	68 (26.2%)	14 (29.8%)	10 (66.7%)
Lack of training in expanded role	11 (4.2%)	1 (2.1%)	--
Lack of confidence in skills	25 (9.6%)	4 (8.5%)	0
Clinical uncertainty	12 (4.6%)	0	1 (6.7%)
Lack of time at work	61 (23.5%)	15 (31.9%)	8 (53.3%)
Lack of performance feedback	2 (0.8%)	2 (4.3%)	--
Lack of motivation to take on new responsibilities	10 (3.8%)	3 (6.4%)	7 (46.7%)
Lack of support from management	14 (5.4%)	4 (8.5%)	4 (26.7%)
Lack of patients presenting with minor ailments	31 (11.9%)	2 (4.3%)	--
Lack of satisfaction with current training and certification program	--	--	2 (13.3%)
Irrelevant to practice	--	--	4 (26.7%)
Other	26 (10.0%)	2 (4.3%)	2 (13.3%)

-- question not applicable and was not asked. Other: respondents were given the option of adding additional barriers in a free text format. These results are explored further in qualitative analysis.
